# Targeting Senescent Cells for a Healthier Aging: Challenges and Opportunities

**DOI:** 10.1002/advs.202002611

**Published:** 2020-10-19

**Authors:** Shuling Song, Tamara Tchkonia, Jing Jiang, James L. Kirkland, Yu Sun

**Affiliations:** ^1^ Key Laboratory of Tissue Microenvironment and Tumor Shanghai Institute of Nutrition and Health Shanghai Institutes for Biological Sciences University of Chinese Academy of Sciences Chinese Academy of Sciences Shanghai 200031 China; ^2^ School of Gerontology Binzhou Medical University Yantai Shandong 264003 China; ^3^ Robert and Arlene Kogod Center on Aging Mayo Clinic Rochester MN 55905 USA; ^4^ School of Pharmacy Binzhou Medical University Yantai Shandong 264003 China; ^5^ Department of Medicine and VAPSHCS University of Washington Seattle WA 98195 USA

**Keywords:** aging, clinical trials, healthspan, senescent cells, senolytics, senotherapy

## Abstract

Aging is a physiological decline in both structural homeostasis and functional integrity, progressively affecting organismal health. A major hallmark of aging is the accumulation of senescent cells, which have entered a state of irreversible cell cycle arrest after experiencing inherent or environmental stresses. Although cellular senescence is essential in several physiological events, it plays a detrimental role in a large array of age‐related pathologies. Recent biomedical advances in specifically targeting senescent cells to improve healthy aging, or alternatively, postpone natural aging and age‐related diseases, a strategy termed senotherapy, have attracted substantial interest in both scientific and medical communities. Challenges for aging research are highlighted and potential avenues that can be leveraged for therapeutic interventions to control aging and age‐related disorders in the current era of precision medicine.

## Graphical abstract

With the trend of global aging, interest is rapidly augmenting in geriatric medicine or geroscience, as manipulation of fundamental aging mechanisms can effectively delay the appearance or reduce the severity of multiple chronic disorders. In such a context, cellular senescence receives considerable attention as an emerging target in prevention or treatment of age‐related diseases, a strategy contributing to healthspan extension.

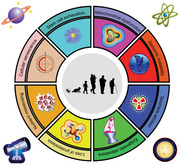

## 1 Introduction

**Figure 1:**
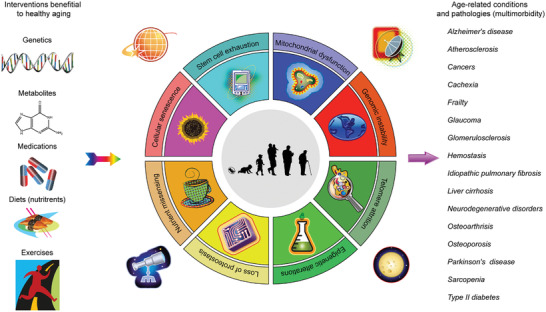
The canonical hallmarks of aging, its accompanying chronic diseases and potential interventions. Aging is characterized by a set of typical hallmarks, including genomic instability, epigenetic alterations, mitochondrial dysfunction, loss of proteostasis, telomere attrition, deregulated nutrient sensing, stem cell exhaustion, and cellular senescence. During natural aging, the incidence of age‐related pathologies increases in a progressive manner, most of which implicate cellular senescence. Therapeutic avenues involving genetic, dietary, or pharmacologic approaches have emerged as potent intervention strategies to prevent or ameliorate aging symptoms, although with different mechanisms. Given that the aged population in a global range is drastically increasing and that aging outstands as the greatest risk factor for the vast majority of chronic diseases responsible for both morbidity and mortality, it is critical, urgent and sagacious to expand geroscience aimed at improving human healthspan. Note, the course of organismal aging is indeed accompanied by various natural activities such as revolution of planets in the universal setting, day and night (exemplified as symbols at the corners of the circle). Part of the figure (mainly the circle) is adapted with permission.^[^ ^164^ ^]^ Copyright year 2020, CellPress.

There is a health challenge increasingly posed by global aging. In the past decades, longer human lives benefiting from improved healthcare and innovative medications have generated a global burden of late‐life pathologies. While survival rates of the elderly population and their mean life expectancy are projected to keep increasing, there is a limit to the maximal life expectancy of humans.^[^ ^1^, ^2^ ^]^ Although health conditions of all ages and consequent benefits are improved, healthspan has not increased yet as remarkably as lifespan.^[^ ^3^ ^]^ Advancing adult age pops up as one of the major risk factors for various chronic disorders such as malignancies, cardiovascular, neurodegenerative, and metabolic diseases.^[^ ^4^ ^]^ Aging impairs cognitive, sensory, and motor function of an individual, while reduced length and severity of late‐life morbidities and their impacts on healthspan are among the most sought aims in civilized societies, an effort termed as “morbidity compression.”^[^ ^5^ ^]^ Notably, some aged people are able to experience illness‐limited healthspan, implying the presence of distinct mechanism(s) supporting such a trait and deserving extensive studies. Omics‐based investigations such as those performed at genetic, epigenetic, protein, and metabolic levels can provide mechanistic insights, together promoting the advancement of aging biology and geriatric medicine (**Figure** **1**). For example, a recent study reported Oximouse, a comprehensive and quantitative mapping strategy to identify the redox‐associated biological processes, uncovered reversible and tissue‐specific modification of protein cysteine residues by reactive oxygen species (ROS), established redox signatures and created networks for myriad proteins in physiology and aging.^[^ ^6^ ^]^ Another omics study applying single‐cell transcriptomic analysis revealed aging‐associated and cell‐type‐specific dysregulation of antioxidative pathways and increased ROS levels in primate ovaries, presenting new diagnostic biomarkers and therapeutic targets for age‐related human ovarian pathologies.^[^ ^7^ ^]^ Clinical investigation of human blood samples from a large cohort comprising young adults and nonagenarians, discovered non‐linear alterations in human plasma proteome with age and identified unexpected signatures and pathways that can offer potential targets for age‐related disorders.^[^ ^8^ ^]^

## 2 The Biology and Impact of Cellular Senescence

In the time course of organismal aging, senescent cells are increasingly accumulated in most, if not all, types of tissues and organs. Originally identified as a tumor suppressor mechanism restricting the expansion of damaged or neoplastic cells, cellular senescence plays indispensable roles in the pathogenesis and exacerbation of a wide spectrum of chronic diseases during aging, defining cellular senescence as a representative example of evolutionary antagonistic pleiotropy.^[^ ^9^, ^10^ ^]^ In the tissue microenvironment (TME), senescent cells mount complex autocrine and paracrine responses by continuously secreting a myriad of extracellular factors including growth factors, cytokines, chemokines, metalloproteases and various extracellular matrix remodeling elements, a feature branded as senescence‐associated secretory phenotype (SASP), or sometimes, senescence‐associated secretome.^[^ ^11^, ^12^, ^13^ ^]^ While these cells are mostly deleterious in later life stages, they do contribute to a subset of physiological events in the organismal lifespan, including a) tissue repair and wound healing via secretion of certain SASP factors, including but not limited to platelet‐derived growth factor AA (PDGF‐AA);^[^ ^14^ ^]^ b) tissue remodeling and regeneration in the context of injury and ageing, such as creation of a permissive TME for in vivo reprogramming via IL‐6 production;^[^ ^15^, ^16^, ^17^ ^]^ c) enhancement of therapeutic vulnerabilities via production of pro‐angiogenic SASP factors such as VEGF, PDGFA/B, and FGF2 to allow tumor vascularization, eventually promoting drug delivery and efficacy of cytotoxic chemotherapy and sensitizing tumors to PD‐L1‐mediated checkpoint blockade;^[^ ^18^ ^]^ and d) embryonic development by promoting morphogenesis of tissues such as the mesonephros and endolymphatic sac of human embryos.^[^ ^19^, ^20^ ^]^ Paradoxically, senescent cells can engage immune surveillance to promote their self‐elimination, a process implicating the recruitment of phagocytic immune cells and mobilization of their nearby progenitors.^[^ ^21^, ^22^ ^]^ However, in the case of persistent damage and/or during natural aging, physiologically autonomous clearance of senescent cells is compromised and dysfunctional cells are accumulated, together generating a chronic pro‐inflammatory microenvironment and contributing to various pathological conditions across the lifespan.^[^ ^23^, ^24^ ^]^

Experimentally described by Hayflick and colleagues in 1960s, cellular senescent was first reported in their studies of human diploid fibroblasts which experienced a limited number of consecutive divisions under culture conditions, a feature later attributed to telomeric attrition.^[^ ^25^, ^26^ ^]^ To date, numerous studies suggest that cellular senescence can occur after exposure of cells to different types of stimuli including DNA damage, epigenetic alteration, proteostasis loss, mitochondrial dysfunction, oncogenic activation, oxidative stress, and even high‐fat diet (HFD) (**Table** **1**).

**Table 1:** A representative list of stimuli and inducers responsible for cellular senescence

Stimulus or inducer	Treatment example or induction route	In vivo consequence
Telomeric attrition	Suppressors of telomerase activity (e.g., SYUIQ‐5,3′‐azido‐3′‐deoxythymidine, pyridostatin)	Aging; cancer^[^ ^123^, ^124^ ^]^
Genotoxic agents	Inducers of DNA replication stress (e.g., bromodeoxyuridine, hydroxyurea); Genotoxic drugs including DNA topoisomerase inhibitors (e.g., etoposide, doxorubicin, mitoxantrone), DNA crosslinkers (e.g., mitomycin C, cisplatin) and drugs with complex effects (e.g., actinomycin D, methotrexate bleomycin)	Cancer regression accompanied by off‐target or side effects^[^ ^32^, ^110^, ^125^, ^126^ ^]^
Deregulated nutrient sensing	Perception of intracellular and/or extracellular nutrient signals (amino acids, glucose, NAD+) by signaling mediated by insulin IGF1, mTOR, or AMPK	Aging‐associated changes^[^ ^127^, ^128^ ^]^
Ionizing irradiation	X‐ray, *γ*‐radiation, UV light	Cancer regression accompanied by side effects^[^ ^125^, ^126^ ^]^
Oncogene activation	HRas, KRas, NRas, BRAF	Cancer progression or suppression^[^ ^129^, ^130^, ^131^ ^]^
Loss of tumor suppressors	p53, PTEN	Tumor inhibition; cancer progression^[^ ^132^, ^133^ ^]^
Oxidative stress	Inducers of reactive oxygen species (e.g., paraquat, hydrogen peroxide)	Aging^[^ ^134^ ^]^
Mitochondrial dysfunction	Decline of mitochondrial malix enzyme 1 (ME1) and malix enzyme 2 (ME2), reduced NAD^+^ level or decreased NAD^+^/NADH ratio	Aging^[^ ^135^, ^136^ ^]^
Loss of proteostasis	ER stress, mTOR activation, UPR events	Aging^[^ ^137^, ^138^, ^139^ ^]^
Suppression by cyclin‐dependent kinase inhibitors	Upregulation of CDKIs such as p16^INK4a^ /p19^ARF^/p21^CIP1^ (downstream of p53) (e.g., nutlin 3a); senescence‐inducing drugs (e.g., abemaciclib, palbociclib, ribociclib)	Tumor suppression; cancer progression^[^ ^140^, ^141^ ^]^
Cytokines	TGF‐*β* and analogs	Aging^[^ ^142^, ^143^ ^]^
Activators of protein kinase C	PEP005, PEP008, TPA/PMA,	Aging^[^ ^144^ ^]^
Epigenetic modifications	DNA methyltransferase suppressors (e.g., 5‐aza‐2‐deoxycytidine); Histone deacetylases inhibitors (e.g., sodium butyrate, trichostatin A); Histone acetyltransferase antagonists (e.g., C646, curcumin); Histone methyltransferases suppressors (e.g., BRD4770)	Aging; cancer^[^ ^145^, ^146^ ^]^
High‐fat diet	Suppression of SIRT1/Beclin‐1/autophagy axis; Accumulation of senescent glial cells in proximity to the lateral ventricle	Diet; insulin resistance; type 2 diabetes; hyperlipidemia; neuropsychiatric disorders (e.g., anxiety‐related behavior)^[^ ^70^, ^147^, ^148^ ^]^
d‐Galactose treatment	Cardiac and mitochondrial dysfunction	Development of obese insulin‐resistance; aging; neuron damage^[^ ^149^, ^150^ ^]^
Autophagy impairment	Loss of specific autophagic programs (e.g., those mediated by p62/SQSTM1)	Aging features and/or age‐related pathological conditions^[^ ^136^, ^137^ ^]^
Lamin B1 reduction	Dysregulation of mTOR and mitochondrial integrity, decrease of DiGeorge syndrome critical region 8 (DGCR8)	Chronic obstructive pulmonary disease (COPD), aging, osteoarthritis, Hutchinson‐Gilford progeria syndrome (HGPS)^[^ ^151^, ^152^ ^]^

Senescent cells are implicated in a wide range of age‐related pathologies, including but not limited to cardiovascular disorders, type 2 diabetes, fibrosis, obesity, malignancies, osteoarthritis, sarcopenia, and neurodegenerative diseases.^[^ ^27^, ^28^ ^]^ Genetic elimination of p16^INK4a^‐expressing senescent cells from experimental mice developing BubR1 deficiency‐associated progeroid and chronic diseases can ameliorate or even revert tissue dysfunction, conferring an extended healthspan to animals.^[^ ^29^ ^]^ Indeed, senescent cell removal provides significant benefits by attenuating multiple pathological conditions, including but not limited to adipose, atherosclerosis, atrophy, cardiomyocyte hypertrophy, cataracts, renal glomerulosclerosis, sarcopenia, tumorigenesis, cancer relapse, osteoarthritis, and tau‐dependent diseases.^[^ ^29^, ^30^, ^31^, ^32^, ^33^, ^34^ ^]^ Removal of senescent cells represents an attractive methodology to counteract aging and age‐related disorders, as it both improves healthspan and extends lifespan.^[^ ^24^ ^]^ Although pharmacologically targeting senescent cells remains a technical challenge for years, recent advancements have evoked the development of senolytics, an emerging class of natural or synthetic molecules that can specifically deplete senescent cells from host tissues. Given the negative impacts of cellular senescence on human vitality and health, industrial pipelines of senolytics hold the potential to provide exciting opportunities for therapeutic intervention.

## 3 Therapeutic Solutions to Target Senescent Cells

Senescence and apoptosis represent distinct cell fates in the context of damage or stress. A number of environmental or inherent stimuli can induce cellular senescence and upregulate expression of a subset of anti‐apoptotic factors, such as Bcl‐2, Bcl‐xL as well as Bcl‐w, a pattern observed at least in senescent cells of some types of tissues.^[^ ^35^, ^36^ ^]^ Thus, beyond cell cycle arrest and the SASP production, another special hallmark of senescent cells is their acquired resistance to damage‐induced apoptosis via engagement of pro‐survival pathways.

### 3.1 First Generation of Senolytics

The intention to develop senolytic drugs first appeared in a pilot study of 2004.^[^ ^37^ ^]^ Experimental manipulations that prolong healthspan and lifespan in mice, caloric restriction (CR) and a *Prop‐1* mutation that reduces growth hormone and other pituitary factors in Ames dwarf mice, delayed accumulation of cells with both p16^INK4a^ upregulation and senescence‐associated *β*‐galactosidase (SA‐*β*‐gal) activity.^[^ ^37^ ^]^ This observation led to efforts to develop senolytic agents, which began in 2004–2005, and also subsequent, independent efforts to generate transgenic mice from which cells with high expression of p16^INK4a^ can be selectively eliminated.

To date, considerable efforts have been made to discover chemical compounds that hold the potential to selectively induce senescent cell death via apoptosis.^[^ ^38^ ^]^ The initial article to report senolytics revealed the involvement of pro‐survival networks (senescent cell anti‐apoptotic pathways, abbreviated as SCAPs), which covers Bcl‐2 family members, ephrins, PI3K isoforms, p21^CIP1^, HIF‐1*α*, and plasminogen activated inhibitors 1 and 2 (PAI‐1 and ‐2), molecules preventing senescent cells from their self‐activation of pro‐apoptotic signaling.^[^ ^36^ ^]^ The contribution of nodes across the SCAP network varies among senescent cell subpopulations of different tissue types. For instance, senescent progenitors of human adipocytes, do not rely on Bcl‐2 family for survival, while their counterparts, senescent endothelial cells do. Dasatinib and Quercetin, the first senolytic drugs, were uncovered by a hypothesis‐driven, mechanism‐based drug screening strategy by using bioinformatics to identify and select agents known to target nodes across the SCAP network. Unlike virtually all other senolytics discovered so far, Dasatinib targets senescent adipocyte progenitors (pre‐adipocytes), a common senescent cell type in older humans as well as patients with diabetes and obesity. Quercetin, a flavonoid, and many other senolytics target senescent endothelial cells of human. In contrast, the combination of Dasatinib and Quercetin (D+Q), once applied together, can specifically target a wider range of senescent cell types than either agent alone.

Shortly after the first “D+Q” report, another 2 senolytics articles demonstrated that Navitoclax (ABT‐263), a potent inhibitor of Bcl‐2, Bcl‐xL, and Bcl‐w, is senolytic against senescent cells of some, but not all tissue types.^[^ ^39^, ^40^ ^]^ Specifically, Navitoclax eliminates senescent myocytes and hematopoietic stem cells, but is largely ineffective for senescent pre‐adipocytes, from sub‐lethally irradiated or naturally‐aged mice.^[^ ^39^, ^40^ ^]^ Navitoclax also depletes senescent cardiomyocytes and restrains profibrotic protein expression in old mice, promoting myocardial remodeling and diastolic function and increasing overall survival post myocardial infarction (MI) in mice.^[^ ^41^ ^]^ Administration of ABT‐737, an inhibitor against Bcl‐xL and Bcl‐w, can efficiently eliminate senescent cells induced by DNA damage in the lungs and those formed in the epidermis by p53/p14^ARF^ pathway activation in transgenic animals.^[^ ^35^ ^]^ In 2017, another article showed that A1331852 and A1155463, specific Bcl‐xL inhibitors, have senolytic efficacy to human umbilical vein endothelial cells (HUVECs) and IMR90 cells, a strain of human lung fibroblasts, albeit not senescent human pre‐adipocytes.^[^ ^42^ ^]^ Interestingly, fisetin, another form of flavonoid, is indeed selectively senolytic to senescent HUVECs, but not IMR90 cells or primary human pre‐adipocytes.^[^ ^42^ ^]^ These data largely substantiated findings reported in early 2015, which suggested Bcl‐xL as a senolytic target in human senescent endothelial cells by RNA interference assays.^[^ ^36^ ^]^

However, special caution should be exercised that Bcl‐2 family suppressors may not be optimal senolytic candidates, mainly due to their potential safety issues. Navitoclax is not specifically senolytic, as it causes apoptosis of several cell types in addition to senescent cells. This agent can deplete foam cells, a type of activated macrophage, from atherosclerotic lesions, hence decreasing disease severity.^[^ ^31^ ^]^ Further, Navitolax causes apoptosis in several other types of non‐senescent cells that are physiologically essential, including neutrophils and platelets, causing potentially life‐threatening neutropenia and thrombocytopenia.^[^ ^43^ ^]^ This raises safety concerns that may preclude the translation of Bcl‐2‐targeting agents into clinical settings. Indeed, though being around for many years, Navitoclax is still not an agent officially approved by the Food and Drug Administration (FDA) of the US, thus not available for physicians to prescribe. In contrast, other drug candidates, such as Dasatinib, which can target SCAPs, have been approved by the FDA since 2006. Overall, Navitoclax appears to be “panolytic” (inducing apoptosis of cells in multiple pathophysiological settings, not limited to senescence) and less truly senolytic than other candidates, such as “D+Q.”

In light of this issue, further efforts have been made to optimize the safety profile of Navitoclax. For instance, a proteolysis‐targeting chimera (PROTAC) technology is used to reduce the platelet toxicity of Navitoclax by converting it into PZ15227 (PZ), a Bcl‐xL PROTAC that integrates Bcl‐xL to the cereblon E3 ligase for degradation.^[^ ^44^ ^]^ As cereblon is poorly expressed in platelets, PZ exhibits less toxicity to platelets with equally or slightly higher potency against senescent cells. In preclinical trials with in naturally aged mice, PZ effectively clears senescent cells and rejuvenates tissue stem and progenitor cells without causing severe thrombocytopenia, thus Bcl‐xL PROTACs may be developed as safer and more potent senolytics than Bcl‐xL inhibitors. Alternatively, galacto‐conjugation of Navitoclax generates a potent senolytic prodrug (Nav‐Gal), which can be preferentially activated by SA‐*β*‐gal activity in multiple cell types.^[^ ^45^ ^]^ Nav‐Gal selectively induces senescent cell death, with a higher senolytic index than Navitoclax, a property associated with reduced activation in non‐senescent cells. Of note, galacto‐conjugation prevents Navitoclax‐induced platelet apoptosis in human and mouse blood samples treated ex vivo, and reduces thrombocytopenia in mouse lung cancer models. A recent study reported a similar prodrug strategy to design a new compound based on the increased SA‐*β*‐gal activity of senescent cells. The prodrug SSK1 can be specifically activated by SA‐*β*‐gal and eliminates mouse and human senescent cells independently of senescence inducers and cell types, thus providing a novel strategy to develop antiaging interventions.^[^ ^46^ ^]^ Together, several lines of novel agents recently emerged and may represent better candidates for future translation into clinical interventions than certain existing senolytics such as Navitoclax, which is associated with substantial hematological toxicity.

### 3.2 Other Senolytics

Beyond the Bcl‐2 family members, there are alternative molecules that are of prominent value as targets for senolytics. HSP90 enhances the survival of senescent cells of some tissue types by stabilizing the cytoplasmic level of p‐Akt, and HSP90 suppressors have recently been found to be senolytic for some senescent cell subpopulations.^[^ ^47^ ^]^ Similar to “D+Q” and Fisetin, the HSP90 inhibitor 17‐DMAG drives clearance of senescent cells, extend of healthspan of animals, and postpone the onset of certain age‐related conditions. Expression of FOXO4, a transcription factor, is upregulated in senescent cells and prevents apoptosis of senescent cells via sequestration of p53 in the nucleus, while a synthetic FOXO4 peptide (namely FOXO4‐DRI) is able to selectively remove senescent cells from the TME niche through p53‐dependent apoptosis.^[^ ^48^ ^]^ The cyclin‐dependent kinase inhibitor p21^CIP1^, a downstream molecule of p53, protects senescent cells of certain tissue origins against death by dampening signal transduction events that engage JNK and caspase in a setting of persistent DNA damage.^[^ ^49^ ^]^

Besides causing apoptosis in non‐senescent cells, Nutlins, a group of small‐molecule antagonists of MDM2, can induce both cellular senescence and apoptosis, especially in the case of glioblastoma multiforme, a most common and invasive form of primary brain tumor.^[^ ^50^ ^]^ Specifically, some Nutlins such as Nutlin‐3a, are cis‐imidazoline analogs that can cause apoptosis across a number of cell types (including non‐senescent cell types) by targeting MDM2‐p53 interactions, resulting in abrogated glioblastoma growth or a dampened SASP.^[^ ^51^, ^52^ ^]^ Intra‐articular administration of a Nutlin, UBX0101, depletes senescent cells in mouse synovium and articular cartilage induced by surgical damage to the knee (US patent 10130628).^[^ ^33^ ^]^ At least in mice, injection of the Nutlin UBX0101 into the knee alleviates post‐surgical osteoarthritis, reducing pain while partially restoring cartilage. However, effects of UBX0101 on pain in a clinical trial of this agent administered into knee joints of patients with femoro‐tibial osteoarthritis were mixed (NCT03513016), and there is hitherto no clear demonstration of senescent cell clearance or radiological improvement in the joints of these patients.^[^ ^53^ ^]^

A recent study indicated that ouabain, a cardiac glycoside (CG), can act as an excellent candidate for senolytics, as senescent cells are efficiently eliminated via apoptosis mediated partially by engagement of the pro‐apoptotic NOXA, a member of the Bcl‐2 superfamily.^[^ ^54^ ^]^ Indeed, senescent cells tend to display a depolarized plasma membrane and harbor increased concentrations of H^+^, features that make them more sensitive to the stimuli of CGs.^[^ ^55^ ^]^ Data from in vivo studies support that CGs are also effective as senolytics in alleviating senescence‐induced lung fibrosis.^[^ ^55^ ^]^ However, CGs can have serious side effects, including cardiac arrhythmias, heart block, and confusion, particularly in the elderly.^[^ ^56^ ^]^

**Figure 2:**
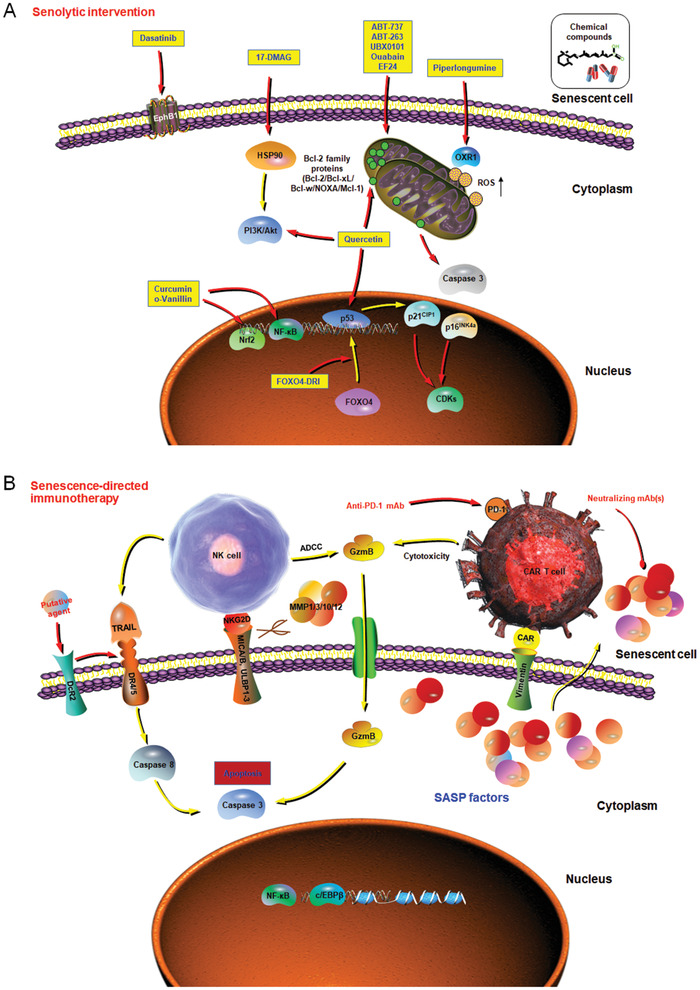
Emerging strategies that showed efficacy in targeting cellular senescence. At late or advanced stages of life, senescent cells progressively accumulate in multiple tissue types and substantially promote the development of multiple age‐related diseases. Increasing lines of studies suggest that targeting specific fundamental mechanisms of aging may be more effective than treating each chronic pathology individually to restrain the impacts of aging on healthspan and lifespan. Pilot trials have been initiated to therapeutically target cellular senescence, such as A) selectively removing senescent cells via induction of their death (senolytic treatment), and B) engagement of the immune surveillance (senescence‐directed immunotherapy), with the potential of each strategy in depleting senescent cells from tissue microenvironment demonstrated by recent investigations. In each mechanistic illustration, yellow arrows mean stimulating or promotive actions, while red arrows represent suppressive or counteractive functions. A) Agents within yellow rectangles are example senolytics. B) Scissors represent shedding of NKG2D ligands by MMPs secreted by senescent cells, especially those induced by oncogene activation. (A) adapted with permission.^[^ ^164^ ^]^ Copyright year 2020, CellPress. We stated “Figure (A) adapted with permission [164]. Copyright year 2020, CellPress.” Please refer to Page 33 (Figure legend, Figure 2).

The curcumin analog, EF24, is a potent senolytic for several types of human senescent cells, such as fibroblasts, endothelial cells, and renal epithelial cells, mainly by reducing the expression of anti‐apoptotic factors Bcl‐xL and Mcl‐1.^[^ ^57^ ^]^ The alkaloid piperlongumine, which derives from long peppers and several medicinal plants, can eliminate senescent human WI38 fibroblasts that arise after exposure to ionizing radiation, replicative exhaustion or oncogenic RAS^G12V^ activation.^[^ ^58^ ^]^ However, pretreatment with Q‐VD‐OPh, a pan‐caspase inhibitor, markedly abrogates piperlongumine‐induced apoptosis of senescent cells, implying involvement of caspase signaling in the senolytic activity of piperlongumine.^[^ ^58^ ^]^ Expression of the antioxidant protein, oxidation resistance 1 (OXR1), which governs the synthesis of many antioxidant enzymes, is elevated in senescent WI38 fibroblasts, but piperlongumine can bind OXR1 directly and enhances its degradation through ubiquitin‐proteasome, thereby forcibly causing cell death^[^ ^59^ ^]^ (**Figure** **2**A).

### 3.3 “D+Q”: The First Senolytics Reported and Clinically Trialed

Combinatorial treatment with the tyrosine kinase inhibitor Dasatinib and the natural flavonoid, Quercetin, effectively induces apoptosis of senescent cells, while their proliferating or quiescent, differentiated counterparts are less responsive.^[^ ^36^ ^]^ Since its FDA approval for clinical use in 2006, Dasatinib is administered daily for treating B lymphoma and certain types of leukemia, along with other diseases in humans. However, other “pan” tyrosine kinase inhibitors, such as Imatinib, are not senolytic. Quercetin, an abundant dietary flavonoid present in apple peels with a bitter taste, selectively interferes with PI3K/AKT and p53/p21^CIP1^/Serpine pathways.^[^ ^60^ ^]^ Mice fed with HFDs develop obesity, hypercholesterolemia, renal tissue senescence and kidney dysfunction, symptoms that can be markedly alleviated upon chronic intervention of Quercetin.^[^ ^60^ ^]^ Combinatorial treatment with “D+Q” selectively eliminates senescent progenitors of human adipocytes and HUVECs in culture. In both mice and humans, the pharmacological half‐life of D+Q is less than 11 h. “D+Q” kill senescent cells in freshly‐established explants from human primary fat tissues within 18 h.^[^ ^61^ ^]^ As it usually take 2–6 weeks for senescent cells to form, which then do not divide, “D+Q” would be as effective if administered to mice intermittently. For example, delivery of these senolytics once a month in a metronomic manner can greatly minimize the risk of side‐effects, despite the short elimination half‐life in vivo.^[^ ^62^ ^]^ Indeed, “D+Q” alleviates senescent cell‐correlated symptoms in multiple pathological conditions, including age‐related frailty and physical dysfunction, idiopathic pulmonary fibrosis (IPF), hyperoxia‐induced reactive airway disease, osteoporosis, insulin resistance, and diabetes, liver steatosis, neuropsychiatric complications of diabetes, and obesity, Alzheimer's disease, cardiac and vasomotor dysfunction, failure of arteriovenous fistulae in hemodialysis patients, and even pre‐eclampsia, a condition promoted by senescent cell accumulation in maternal blood vessels^[^ ^36^, ^60^, ^61^, ^62^, ^63^, ^64^, ^65^, ^66^, ^67^, ^68^, ^69^, ^70^, ^71^, ^72^, ^73^, ^74^, ^75^, ^76^ ^]^

In the first ever clinical trial of senolytics, “D+Q” showed decent efficacy in improving physical function of patients with IPF, a relentlessly progressive, lethal, and cellular senescence‐related lung pathology for which to date there have been only limited treatment options.^[^ ^77^ ^]^ The interim report from a recent clinical trial of “D+Q” in patients with advanced diabetes with kidney disorder indicated that this senolytic combination is optimal in minimizing the burden of senescent cells in vivo.^[^ ^78^ ^]^ Analyses of adipose tissue biopsies before the onset of and 11 days after the completion of a 3 day treatment in that trial with oral “D+Q” suggested a significant decrease of senescent cells with p16^INK4a^‐expression and SA‐*β*‐gal activity. “D+Q” also markedly restricted macrophage infiltration in adipose tissues of these patients and avoided the development of “crown‐like structures,” indicators of inflammation and fibrosis, with levels of a composite of 10 circulating SASP hallmark factors such as IL‐1*α*, IL‐6, FGF2, GM‐CSF, and MMPs (2/9/12) remarkably lowered in patient plasma.

Given the inspiring data from these clinical trials, development of pipelines to identify senolytic drugs followed by translation into clinical settings is highly valued by both industrial and scientific communities. However, there are caveats. The impact of “D+Q” on the treatment of hepatocellular carcinoma (HCC) was recently explored, and the preliminary data suggest that maximal cytostatic doses for D and/or Q (1 + 1 µm) failed to show efficacy in removing doxorubicin‐induced *β*‐gal‐positive senescent cells.^[^ ^79^ ^]^ Moreover, “D+Q” did not affect flattened morphology, p16^INK4a^ activation, the SASP expression or *γ*H2AX focus formation of senescent cells, suggesting these compounds may be ineffective when combined with certain types of senescence‐inducing chemotherapy, especially against experimental HCC.

Although Dasatinib is an orally bioavailable synthetic small molecule compound against Src‐family tyrosine kinases that are clinically administered for chronic myelogenous leukemia, this agent can induce side‐effects such as cardiovascular events, myelosuppression, fluid retention, pulmonary arterial hypertension, severe dermatologic reactions and cell lysis syndrome, at doses and frequency used for anticancer effects.^[^ ^80^ ^]^ However, it remains unknown how these complications will correlate with the intermittent and infrequent administration of dasatinib for senescent cell elimination in prospectively expanded clinical studies. In a similar case, several lines of data suggest that quercetin has contraindications with respect to potential drug‐drug interactions,^[^ ^81^, ^82^ ^]^ and it is largely open whether these possible adverse events will be observed in future senolytic trials that comprise intermittent dosing regimens with patients developing age‐related diseases.

### 3.4 Immune‐Potentiated Approaches

In contrast to aforementioned intrinsic pathway‐directed depletion of senescent cells, therapeutic approaches mediated by the immunity represent a different strategy that is attracting substantial attention. Senescent cells are immunogenic per se and are subject to immune surveillance.^[^ ^22^, ^83^ ^]^ A natural but distinct mechanism responsible for dampened inflammatory response during cellular senescence (particularly replicative senescence and therapy‐induced senescence) and aging in Spalax was recently discovered, providing a rationale for developing senolytic agents that act through the immune system.^[^ ^84^ ^]^ Although senescent cells can chemo‐attract various immune cell types, a process promoting senescent cell clearance by the immune components, many studies have suggested that senescent cells can also circumvent innate and adaptive immune responses. Therapeutics enhancing immune‐potentiated clearance of senescent cells include but are not limited to vaccines and small molecule immune modulators.^[^ ^85^ ^]^

Senescent cells including those induced by replicative exhaustion, oncogene activation and DNA‐damaging agents, display enhanced expression of ligands for NKG2D, a homodimeric C‐type lectin‐like receptor present on the surface of NK and cytotoxic T cells.^[^ ^86^ ^]^ Thus, NKG2D ligands may empower immune‐mediated clearance of these cells. Mechanisms of senescent cell surveillance can be context‐dependent. Expression of the oncogene NRAS^G12V^ induces senescence in hepatocytes, generating a combined phenotype of both innate and adaptive immune responses, whereby CD4+T cells cooperate with both monocytes and macrophages to clear senescent hepatocytes.^[^ ^22^ ^]^ However, senescent cells can evade immune recognition via engagement of MMP‐dependent shedding of NKG2D ligands such as MICA/B and ULBP1‐3, thus disabling NKG2D‐mediated immune surveillance in a paracrine manner.^[^ ^87^ ^]^ Although expressing more soluble MICA/B ligands than replicatively senescent or therapy‐induced senescent cells, oncogene RAS^G12V^‐induced senescent cells evade NKG2D receptor‐mediated immune surveillance by expressing high levels of MMP1/3/10/12, a group of SASP factors that have protease activities.^[^ ^87^ ^]^ Thus, immune evasion and persistence of RAS^G12V^ senescent cells is mediated, at least in part, by active protease secretion and high levels of NKG2D Ligand shedding.

Such coordinated immune editing also takes place in residual, drug‐resistant tumors from breast and prostate cancer patients after genotoxic chemotherapy, which frequently induces cellular senescence in damaged tissues,^[^ ^87^ ^]^ exemplifying how senescent cells can elude immune surveillance and implying the necessity of senolytic strategies to enhance clearance of senescent cells arising after clinical treatment or accumulating in aged tissues. Alternatively, administration of polyI:C, an immune booster, helps promote elimination of senescent cells mediated by NK cells in fibrotic livers, facilitating resolution of fibrosis induced by acute tissue damage.^[^ ^88^ ^]^ Nevertheless, in vivo treatment with a strong immune stimulator may likely overwhelm the immune system in an aged individual, such as the case of cytokine storm observed in patients developing severe COVID‐19 and manifesting acute respiratory distress syndrome, the leading cause of mortality among those affected in current global range.^[^ ^89^, ^90^ ^]^ Thus, clinically safer and more precise immune modulators need to be pursued for this purpose.

Beyond immune‐boosting therapeutic strategies, an augmenting arsenal of tools including chimeric antigen receptor T cells, present the avenue to facilitate redirecting immune responses against senescent cells.^[^ ^91^ ^]^ However, a set of highly selective senescence biomarkers is not yet available that can allow this strategy to be pursued. Senescent cells are partially protected from NK cell‐mediated cytotoxicity via upregulation of decoy receptor 2 (DcR2), a member of the tumor necrosis factor‐related apoptosis‐inducing ligand (TRAIL) receptor family.^[^ ^92^ ^]^ DcR2 suppresses activation of death receptors 4 and 5 (DR4/5) caused by TRAIL, confining cell death to perforin‐ and granzyme‐mediated signaling.^[^ ^93^ ^]^ Thus, searching for ways to promote these activities, such as shielding DcR2, might confer senescent cells with increased vulnerability to innate immune surveillance. Although technical advances regarding immune‐mediated senescent cell clearance are still preliminary and await continued investigation, there appears charming potential for harnessing the immune system to target senescent cells.^[^ ^28^ ^]^ Identification of specific and novel senescent cell surface markers might accelerate the development of immune‐associated senotherapies (Figure 2B).

### 3.5 Senomorphics or Senostatics

Rather than directly killing senescent cells, some agents including plant‐derived compounds and dietary supplements can restrain senescence‐associated phenotypes by specifically suppressing the SASP or pro‐inflammatory secretome, thus minimizing the impact of senescent cells in the tissue microenvironment such as development of paracrine senescence. Examples for this category of “senomorphics” (or alternatively, “senostatics”) include but are not limited to apigenin, kaempferol, glucosamine, 4,4′‐dimethoxychalcone, nordihydroguaiaretic acid and resveratrol.^[^ ^94^, ^95^, ^96^, ^97^, ^98^, ^99^ ^]^ To date, there are two different types of “senomorphics,” including one that controls the SASP regulatory network and the other specifically against a certain component of the SASP. For the former, a group of therapeutic targets have already emerged as key nodes of the SASP signaling network and were mechanistically dissected, including but not limited to NF‐*κ*B, p38MAPK, GATA4, mTOR, BRD4, and TAK1, thus providing optimal targets to develop effective solutions.^[^ ^100^, ^101^, ^102^, ^103^, ^104^, ^105^, ^106^ ^]^ In contrast, the second type of senomorphics has not been seriously considered yet, mainly due to the fact that the SASP composition highly varies, depending on different cell types, senescence stages, and senescence‐inducing stimuli,^[^ ^107^, ^108^ ^]^ although elimination of a single factor of the full spectrum of SASP has demonstrated efficacy in some situations.^[^ ^109^, ^110^, ^111^ ^]^ While further investments such as categorization of SASP factors according to their functions in a context‐dependent manner may be a daunting challenge, such efforts are important in a long run, as they can pave the road to suppress deleterious effects of the SASP while keeping its beneficial functions.

However, most senomorphics share a potential drawback. In contrast to senolytics, which deliver cytotoxicity to senescent cells when given in an intermittent manner, senomorphics may need to be taken regularly, or even consecutively, for an extended period to show maximal benefits. Despite such a technical limitation, consumption of diet enriched with phytochemicals from fruits and/or vegetables may provide considerable health benefits, at least by improving stress resistance and inducing antiaging effects. Although it remains unclear whether senomorphics are generally beneficial to human patients, individuals regularly consuming d‐glucosamine or metformin tend to live longer than their counterparts,^[^ ^112^, ^113^ ^]^ largely consistent with the antiaging capacity of these agents (**Table** **2**).

**Table 2:** Examples of agents that show senolytic or senomorphic activity

Agent	Source	Target (s)	References
***Senolytics***			
ABT263 (Navitoclax)	Synthetic	Bcl‐xL, Bcl‐2, Bcl‐W	^[^ ^39^, ^40^ ^]^
ABT737	Synthetic	Bcl‐xL, Bcl‐2, Bcl‐W	^[^ ^35^ ^]^
A1331852	Synthetic	BCL‐xL	^[^ ^42^ ^]^
A1155463	Synthetic	BCL‐xL	^[^ ^42^ ^]^
UBX0101	Synthetic	MDM2	^[^ ^33^ ^]^
Ouabain	Natural (plants)	Na^+^/K^+^‐ATPase	^[^ ^54^ ^]^
Cardiac glycosides (Proscillaridin A, Digoxin, etc.)	Natural (plants)	Na^+^/K^+^‐ATPase	^[^ ^153^ ^]^
Quercetin	Natural (Vegetables, dietary Supplements)	PI3K/Akt, p53	^[^ ^36^, ^67^, ^72^, ^76^ ^]^
Dasatinib	Synthetic	Src family tyrosine kinases (BCR/ABL, Src, c‐Kit, ephrin)	^[^ ^36^, ^67^, ^72^, ^76^ ^]^
Curcumin	Natural (plants)	Nrf2, NF‐*κ*B (p65)	^[^ ^154^ ^]^
*o*‐Vanillin (curcumin metabolite)	Natural (plants)	Nrf2, NF‐*κ*B (p65)	^[^ ^154^ ^]^
EF24 (curcumin analog)	Synthetic	Bcl‐xL, Mcl‐1	^[^ ^155^ ^]^
Fisetin	Natural (plants, fruits, dietary supplements)	Sirtuins	^[^ ^156^ ^]^
Piperlongumine	Natural (piper plants)	Caspase 3	^[^ ^58^ ^]^
FOXO4‐DRI peptide	Synthetic	FOXO4‐p53 interaction	^[^ ^48^ ^]^
Alvespimycin (17‐DMAG)	Synthetic (geldanamycin derivative)	HSP90	^[^ ^47^ ^]^
***Senomorphics***			
Apigenin	Natural (plants, fruits, vegetables, tea)	NF‐*κ*B	^[^ ^94^ ^]^
Kaempferol	Natural (fruits, vegetables)	NF‐*κ*B	^[^ ^94^ ^]^
Glucosamine	Natural (Dietary supplements)	ROS, p21^CIP1^, autophagy	^[^ ^157^, ^158^ ^]^
Nordihydroguaiaretic acid (NDGA)	Natural (Creosote bush)	p300 acetyltransferase	^[^ ^97^ ^]^
Resveratrol	Natural (fruits, red wine, dietary supplements)	SIRT1	^[^ ^159^, ^160^ ^]^
Rapamycin	S. hygroscopicus	mTOR	^[^ ^103^, ^104^ ^]^
Metformin	Synthetic	AMPK	^[^ ^161^, ^162^ ^]^
Ruxolitinib	Synthetic	JAK1/2	^[^ ^163^ ^]^
SB203580	Synthetic	p38MARPK	^[^ ^101^ ^]^
5Z‐7‐oxozeaenol	Synthetic	TAK1	^[^ ^106^ ^]^

## 4 Concluding Remarks and Future Perspectives

Aging a complex and dynamic process that drives progressive decline of tissue functionality, physiological integrity and its regenerative potential. Demographic data suggest that the aging rate of global populations continues to rise, a tendency resulting in the prevalence of various chronic pathologies and production of public health epidemics.^[^ ^114^, ^115^ ^]^ Although preclinical studies convincingly support that senescent cell elimination can ameliorate or even revert the pathological progress of multiple diseases, there are challenges that need to be well taken to ensure successful translation of interventional strategies.

Senescent cells are increasingly abundant within tissues in the course of aging. Despite such a fact, cell senescence is essential in certain physiological aspects such as tumor suppression, tissue repair, and regeneration, wound healing, vascular remodeling and embryonic development in a damage‐independent manner.^[^ ^14^, ^15^, ^18^, ^20^, ^116^, ^117^ ^]^ Thus, unselectively manipulating cellular senescence during these processes may compromise physiological integrity and affect patient health. Although the effect of senolytics on individual physiological processes remains largely unexplored in the experimental context, current senolytics generally induce apoptosis of senescent cells upon a transient exposure, with their administration limited to a short period of time to avoid ensuing side‐effects.

Further, the targeting specificity of senolytics represents a challenging issue. There are possible strategies to overcome this difficulty and optimize selectivity. First, a feasible solution is to develop pharmacologically active compounds and probes already tested in preclinical studies by exploiting synthetic lethal vulnerabilities, such as the drug delivery system taking advantage of the increased lysosomal *β*‐galactosidase activity of senescent cells to develop agents including galacto‐oligosaccharides and the recently reported senescence‐specific killing compound 1 SSK1, wherein the acetyl group and *β*‐gal‐responsive group improves cellular permeability and specificity.^[^ ^46^, ^118^ ^]^ Second, an alternative option for enhancing specificity is to modify the therapeutic or diagnostic agent that can be activated by external stimuli or enzymatic actions, as exemplified by the case of galactose‐based nanoparticles, encapsulation methods and probes utilizing *β*‐galactosidase expression of senescent cells.^[^ ^118^, ^119^, ^120^ ^]^ Third, expression of the SASP components by senescent cells might be employed to stimulate drug delivery systems and inactive pro‐senolytics or synthetic probes, for in vivo imaging purposes including magnetic resonance imaging and positron emission tomography.^[^ ^27^ ^]^ Synthetically lethal compounds would be loaded into nanoparticles targeting senescent cells, thereby extensively widening their therapeutic windows, efforts requiring a more detailed understanding of the senescent cell‐specific signaling pathways or regulatory networks. For future clinical studies, there is still a major need to develop a set of novel, accurate and noninvasive senescence biomarkers for reliable assessment the burden of cellular senescence in the TME. Patient candidates for senotherapies will be appropriately evaluated according to their senescent burden, an essential step ensuring interventional necessity and clinical success.

Given the heterogeneity of clinical patients and the complexity nature of aging and age‐related disorders, future interventions against cellular senescence will likely be context‐dependent, or more accurately, personalized. In response to the urgent and unmet clinical, healthcare, workforce, and economic needs of global aging populations who are suffering from aging‐related conditions and multimorbidity, the world health organization (WHO) has recently called for a public health attention based on the law of human rights.^[^ ^121^ ^]^ To conduct a clinical trial, a corresponding disease classification code is necessary and can be adopted according to the WHO international classification of diseases guideline.^[^ ^122^ ^]^ A comprehensive and systematic staging system would allow classification of aging individuals from the point of absence of organ and tissue senescence pathology and any appearance, features, and diagnostic criteria. Such a proposed framework is intended to be applied independently or combined with currently present classification codes in a complementary pattern, across disease diagnosis, prevention, and clinical intervention.^[^ ^122^ ^]^ In the long run, the success of any clinical management to counter multimorbidity will be affected by the wish of an elderly individual to minimize its effects, and correspondingly, his/her compliance with preventative measures. For those willing, however, lifestyle improvement protocols and preventative interventions are handily available, with a line of emerging and promising regimens on the new horizon of precision medicine.

**Figure 3:**
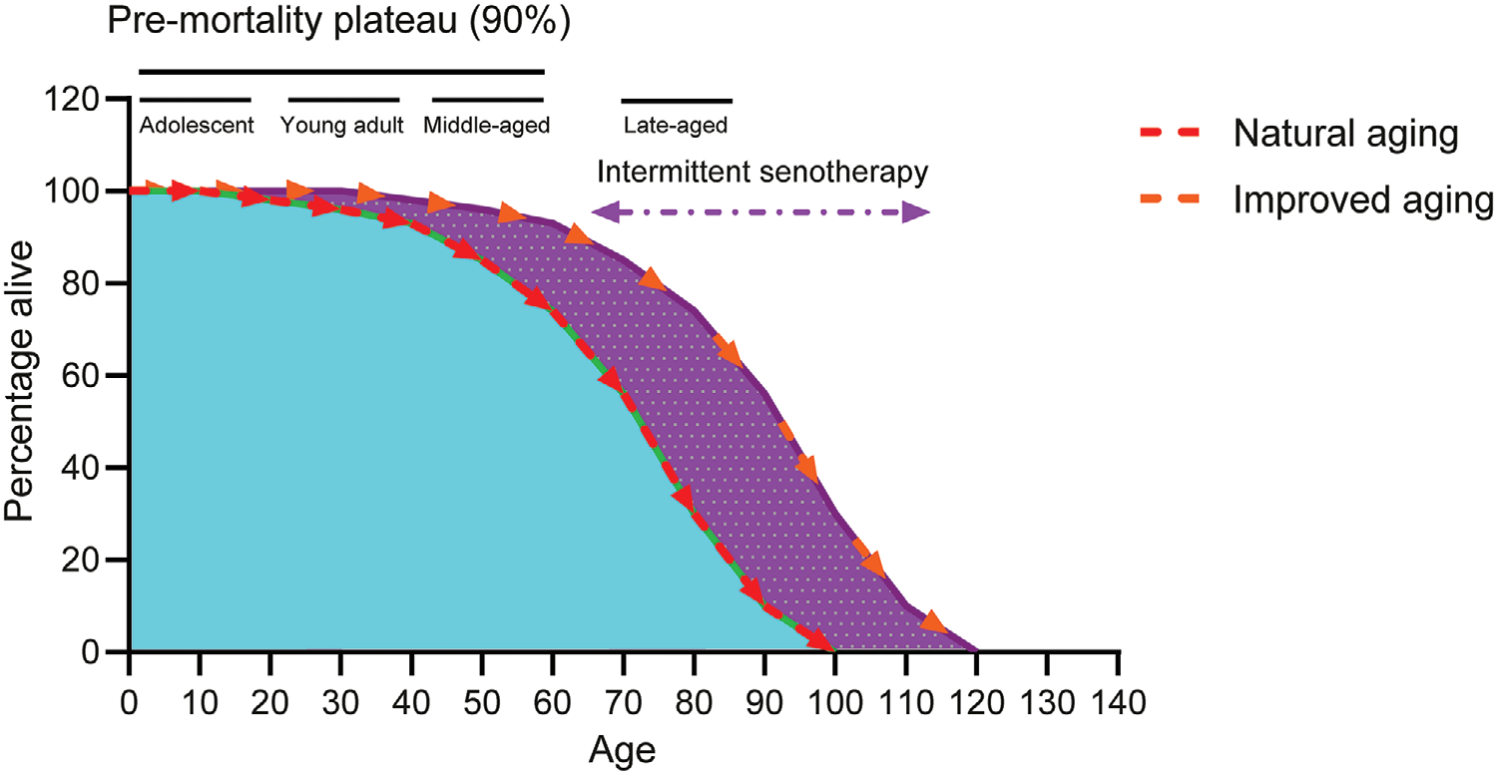
A hypothetic model to illustrate senotherapy‐mediated healthy aging. The first stage of the survival curve refers to the pre‐mortality plateau phase (PPP), during which ≈10% of the overall population is lost over a relatively long period. Alternatively referred to as a healthy lifespan, the PPP covers three sub‐stages, including an adolescent period when the body actively develops, a young adult period when the individual is physiologically mature, and a middle‐aged period when the mortality rate begins to increase. During the overall PPP span, 90% of the population remains alive, after which the mortality rate significantly arises and the population declines rapidly, a period constituting the late‐aged phase. However, in the case of senotherapy which theoretically comprises multiple waves of medications with senolytics administered in an intermittent or metronomic manner, the lifespan can be remarkably extended. Senotherapy can eliminate senescent cells, promote tissue repair, improve organ regeneration, and ameliorate multiple age‐related pathologies, thus providing a prominent avenue for a healthier aging of human populations.^[^ ^165^, ^166^ ^]^

Despite a limited number of early phase clinical trials on senotherapies so far, we predict with enthusiasm a rapid expansion in the translational field of aging biology in next years, as accompanied by blooming techniques and advancing concepts in geriatric medicine (**Figure** **3**). Together, we are entering an exciting era of geroscience, and novel therapies toward medical applications will have substantial impacts on human health and longevity.

## Conflict of Interest

J.L.K. has a financial interest related to this research. Patents on senolytic drugs are held by Mayo Clinic. This research has been reviewed by the Mayo Clinic Conflict of Interest Review Board and was conducted in compliance with Mayo Clinic Conflict of Interest policies.
